# Is Behavioral Tolerance Learned?

**Published:** 1997

**Authors:** Muriel Vogel-Sprott

**Affiliations:** Muriel Vogel-Sprott, Ph.D., is a professor in the Department of Psychology at the University of Waterloo, Waterloo, Ontario, Canada

**Keywords:** AOD tolerance, reinforcement, AOD impairment, AODE (alcohol and other drug effects), behavior, AOD intoxication, expectancies, learning, context dynamics, literature review

## Abstract

Both scientific and anecdotal evidence indicates that social drinkers can develop resistance (i.e., behavioral tolerance) to alcohol’s impairing effects over time. Although repeated exposure to alcohol is thought to explain tolerance development on a physiological level, the acquisition of behavioral tolerance appears to involve additional factors. In particular, learned associations between a drinker’s behavior following alcohol consumption and the subsequent consequences may play an important role. When favorable consequences result from displaying unimpaired (i.e., tolerant) behavior after drinking, a drinker learns to develop behavioral strategies to compensate for alcohol’s effects. In contrast, if a drinker does not receive a reward for unimpaired behavior—or finds that a more favorable outcome follows the display of intoxicated behavior—tolerance does not develop. Studies show that subjects also can develop behavioral tolerance to alcohol when they practice a task while impaired by factors other than alcohol or when they mentally rehearse task performance while under the influence of alcohol.

The term “tolerance” refers to a reduction in the intensity of the effect of alcohol (or other drugs) over the course of repeated use. Thus, a person developing tolerance to alcohol must drink greater quantities of alcoholic beverages to produce the same effect that had been previously achieved at a lower consumption level. This phenomenon may reflect the acquisition of tolerance to alcohol’s physiological effects (e.g., reduced body temperature) as well as its behavioral effects (e.g., impaired motor coordination). Researchers generally believe that the physiological actions of alcohol and other drugs contribute to tolerance by triggering the body to produce opposite physiological reactions in an effort to compensate and restore stable internal conditions (i.e., homeostasis). A compensatory response usually is assumed to become stronger each time a person uses alcohol or other drugs and to subside gradually during a period of abstinence.

Research on the effects of alcohol on cellular and neuronal functions has provided much information on where and how alcohol acts in the brain to produce its physiological effects. In contrast, no direct causal relationship has been established between any specific biological change induced by alcohol exposure and a particular behavioral response (e.g., Hunt 1993). Nonetheless, clinical observations suggest that tolerance plays an important role in alcohol abuse and addiction. For example, compared with social drinkers,^1^ alcoholics have more exposure to alcohol, giving their bodies repeated opportunities to activate increasingly strong compensatory responses. In addition, alcoholics may display remarkable resistance (i.e., tolerance) to the effects of drinking alcoholic beverages in quantities that would greatly impair social drinkers. These observations accord with the notion that apparent sobriety after drinking or an “ability to handle one’s liquor” (i.e., exhibit tolerance to alcohol’s behavioral effects) may be a useful pathological diagnostic symptom (American Psychiatric Association 1994).

The transition from social to abusive drinking often occurs gradually over years of drinking sessions, however, and only in some drinkers. This finding suggests that the acquisition of behavioral tolerance to alcohol in social drinkers results from factors in addition to the extent of alcohol exposure.

One research-supported explanation involves environmental events that predict drug administration. Investigators have found that the expectation of receiving a drug can affect tolerance. For example, studies have shown that animals made tolerant to a drug through repeated administrations in a distinctive setting subsequently will display greater tolerance when the drug is administered in this same setting as opposed to a new one (see, for example, Siegel 1989). Researchers have interpreted these results in terms of associative learning: When distinctive events reliably precede drug administration, they serve as a signal that provides a basis for expecting the drug. When tolerance is established, this expectation results in anticipatory compensatory reactions to reduce the drug’s effect. Consequently, “tolerance is maximally displayed following ‘expected’ drug administration but not following ‘unexpected’ drug administration” (Siegel 1989, p. 116).

People likely acquire tolerance to alcohol’s behavioral effects in drinking situations where reliable cues, such as liquor bottles, signal alcohol availability. Research indicates that when alcohol is expected and received, however, a social drinker may demonstrate behavioral tolerance while performing one task but not another (see, for example, Vogel-Sprott 1979). Alcohol expectations arising from events that precede drinking therefore do not appear to fully account for the development of behavioral tolerance in social drinkers. Thus, the question remains: What additional factors in drinking situations affect the acquisition of tolerance to alcohol’s behavioral effects among social drinkers?

This article focuses on an intriguing potential factor currently undergoing investigation in behavioral neuroscience research—the idea that environmental events associated with a drinker’s behavior following alcohol consumption (e.g., positive or negative consequences) play a role in determining whether the drinker will develop behavioral tolerance (see box, p. 167). This research has been guided by the theory that environmental events known to affect the learning of new behavior likewise will influence behavioral tolerance to alcohol.^2^ This article first presents some historical background for theories on the development of behavioral tolerance, then reviews contemporary findings and their implications. (For a more detailed discussion of this work, see Vogel-Sprott 1992.)

**Contributors to Tolerance Development**

*Physiological compensation.* Alcohol consumption induces physiological reactions to compensate for its effects; these reactions strengthen with repeated alcohol exposure and contribute to tolerance.

*Learned expectation of receiving alcohol.* Reliable repetition of specific events preceding alcohol consumption leads to the expectation of receiving alcohol; these expectations result in compensatory reactions that contribute to tolerance.

*Learned expectation of behavioral consequence.* Favorable events reliably associated with sober behavior lead to expectations of a positive consequence for such behavior; these expectations result in the development of behavioral strategies to overcome alcohol-induced impairment and contribute to tolerance.

## Historical Background

Although the view that tolerance stems from alcohol-induced compensatory reactions is prevalent in the scientific literature, other possibilities have received attention. The idea that environmental events following alcohol consumption contribute to tolerance development has roots in the 19th century, when opinion held that tolerance was largely under volitional control. For example, more than 100 years ago, MacNish penned the following observation:

The mind exercises a considerable effect upon drunkenness, and may control it powerfully. When in the company of a superior whom we respect, or of a woman in whose presence it would be indelicate to get intoxicated, a much greater portion of liquor may be withstood than in societies where no such restraints operate (MacNish 1832, p. 45).

MacNish’s notion that circumstances following drinking can affect behavioral tolerance has been advanced during the 20th century as well. Goldberg (1943) speculated that “psychic compensation” contributed to the tolerance displayed by alcoholics. A few decades later, Dews (1962) proposed that drinkers’ tolerance might result from new learned behavior that compensates for alcohol’s impairing effects. In addition, MacAndrew and Edgerton (1969) noted that drinking orgies in aboriginal cultures resulted in gross intoxication in some societies but tolerance in others; they attributed this variance to learned conformity to different culturally specific standards of behavior under alcohol.

Anecdotes about grossly inebriated drinkers who become sober when they believe it is important to do so also suggest that events after drinking alcohol can affect tolerance. This ability of drinkers to “sober up” apparently provided the impetus for introducing the breathalyzer to measure blood alcohol concentration (BAC) levels for forensic purposes. Before the breathalyzer was available, a medical examination of suspected intoxicated drivers was required to support the charge. Goldberg and Havard (1968) reported that these clinical assessments were completely unreliable. They noted that suspects faced with a doctor called in by the police often were capable of “pulling themselves together” to pass all the clinical tests. After the suspect satisfied the police physician and was free to leave, however, signs of intoxication reasserted themselves. As a result, the police frequently had to assist suspects from the station and escort them home.

Tolerance on the part of intoxicated suspects appears to depend on the expectation of a reward for sober behavior. The suspects exhibited sober behavior (i.e., tolerance) when they perceived a payoff for doing so but did not act sober in the absence of a rewarding consequence for compensating for alcohol’s effect. In this respect, tolerance resembles a goal-directed (i.e., instrumental) learning response that becomes dominant when associated with a reward and extinguishes when the reward is withdrawn.

## Effects of Rewarding Sober Behavior

Using simple and complex psychomotor tasks as tests, researchers have studied the effect of rewarding sober performance on the development of alcohol tolerance. Motor-skill tasks, such as tracking randomly presented visual targets, typically require eye-hand coordination of complex responses. Alcohol-induced impairment of a psychomotor skill is characterized by reduced accuracy, slower performance, or both. In studies using motor-skill tasks, groups of social drinkers learn to perform a task, then typically attend three or four weekly drinking sessions in which they receive 0.62 gram of alcohol per kilogram of body weight. (For a person weighing 154 pounds, this dose equals approximately three 12-ounce bottles of 5-percent beer.) During each 21_/2_-hour session, the subjects perform a task at regular intervals as their BAC rises to a peak (to approximately 0.08 percent, which is the BAC limit for drivers in at least 14 States) and subsequently declines. In these types of studies, researchers determine alcohol’s impairing effect by measuring how much the subjects’ performance differs from their baseline proficiency on the task while sober. Each week of the study’s duration, the researchers measure the subjects’ average impairment under the alcohol dose and assess their development of tolerance by noting any reduction in impairment as the alcohol dose is repeated over time.

**Figure 1:**
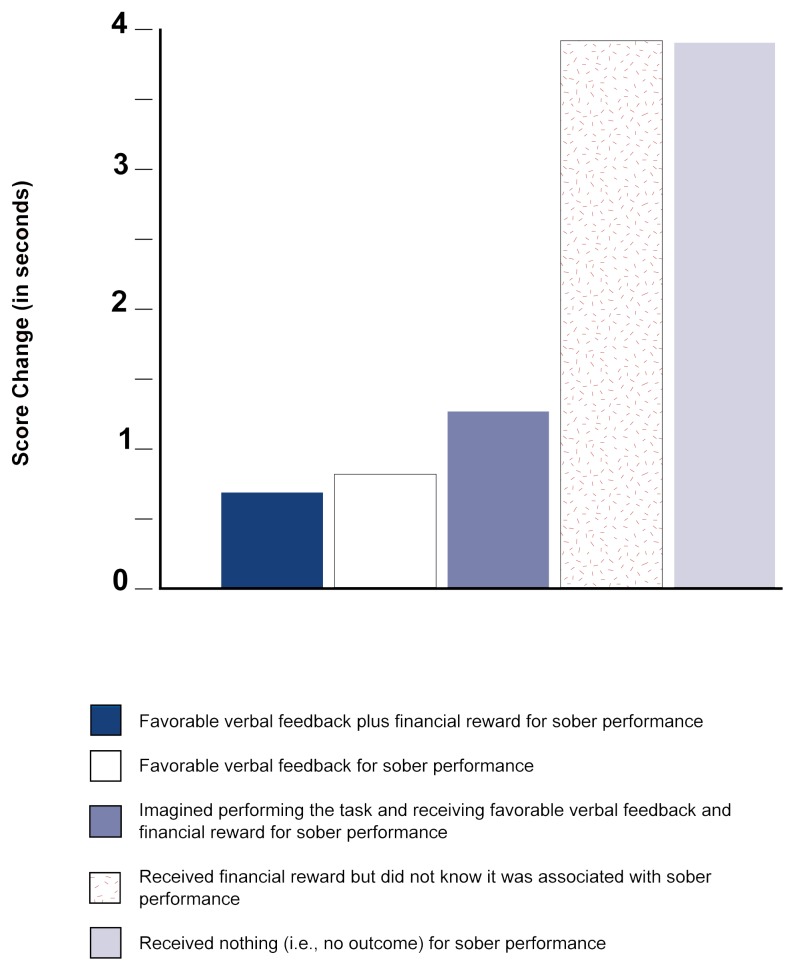
Alcohol-induced impairment of task performance. Completion times for subjects’ sober performance of an eye-hand coordination task were obtained and used as baseline scores. Subjects then had a chance to overcome impairment (i.e., build tolerance) during three sessions of alcohol consumption followed by either performing or mentally rehearsing the task. Next, all subjects performed the task after drinking. This graph shows the difference for each group between the average time needed to complete the final task and the average baseline score. Subjects in groups receiving actual or imagined rewards during previous sessions displayed greater tolerance by achieving times closer to their sober performance (i.e., low score changes). Results indicate that actual or mental practice, when associated with a reward for unimpaired behavior, enhances behavioral tolerance. NOTE: Zero = Sober baseline score. SOURCE: Adapted from Sdao-Jarvie, K., and Vogel-Sprott, M. Learning alcohol tolerance by mental or physical practice. *Journal of Studies on Alcohol* 53(6):533–540, 1992. p. 538.

One way to reward sober behavior is to pay drinkers whenever their performance while intoxicated matches their level of achievement while sober. In studies employing this reward treatment, researchers have observed a progressive development of tolerance with repeated drinking sessions (e.g., Beirness and Vogel-Sprott 1984). In addition, they have found that no such increase in tolerance occurs under control conditions in which the reward is unrelated to sober performance. Although social drinking situations ordinarily do not pay drinkers when they act sober, such behavior might result in verbal approval from others (e.g., the comment “good”). Some studies have compared the rewards of monetary payment versus more realistic verbal approval for sober performance in terms of their tolerance-inducing effects (e.g., Sdao-Jarvie and Vogel-Sprott 1991). Interestingly, these studies have found that both forms of reward have similar efficacy. In general, a favorable outcome for sober behavior appears to enhance the development of tolerance in social drinkers, and different types of favorable outcomes can accomplish the same end (see figure 1 for the results from one study).

In contrast, behavioral tolerance disappears when the reward is withheld (Mann and Vogel-Sprott 1981; Zack and Vogel-Sprott 1993). This finding is consistent with learning studies showing that subjects trained to produce a particular response for an immediate reward will extinguish this response readily when the reward is withheld. After subjects had acquired tolerance during drinking sessions in which they were immediately rewarded for sober performance, for example, their tolerance vanished when the reward was withheld on subsequent drinking sessions, even though they continued to consume the same amount of alcohol (Mann and Vogel-Sprott 1981; Zack and Vogel-Sprott 1993).

A drinker may readily discriminate between the presence or absence of an immediate reward for sober performance, but this circumstance is unlikely to characterize social drinking situations. More commonly, behavior after drinking may result in a significant consequence or outcome only when such behavior deviates from a socially accepted standard (e.g., when behavior is obnoxious). In these situations, the consequence is likely to be negative. Therefore, the absence of an unfavorable consequence serves as a reward for displaying tolerance as sober behavior. Withdrawal of the reward, however, means that nothing happens (i.e., no unfavorable consequence ensues), even though the drinker meets the socially accepted standard of behavior. Consequently, the drinker cannot distinguish the presence of a reward from its absence, because both conditions are characterized by the lack of an aversive consequence.

Learning studies indicate that rewarding a response by withholding an aversive consequence results in the acquisition of a persistent response that is difficult to extinguish when the reward is removed. Researchers have obtained similar results using this procedure to develop behavioral tolerance through repeated doses of alcohol: In a study by Zack and Vogel-Sprott (1995), subjects performing a task received an unfavorable verbal consequence (e.g., the comment “bad”) when their performance deviated from a sober standard of behavior (i.e., the subjects’ alcohol-free level of proficiency on the task). The absence of this consequence served as a reward when they matched the standard. Such training encouraged the subjects to act sober (i.e., it enhanced their behavioral tolerance to repeated doses of alcohol). Furthermore, the subjects retained this tolerance well during subsequent drinking sessions in which all consequences for performance were withheld.

**Figure 2:**
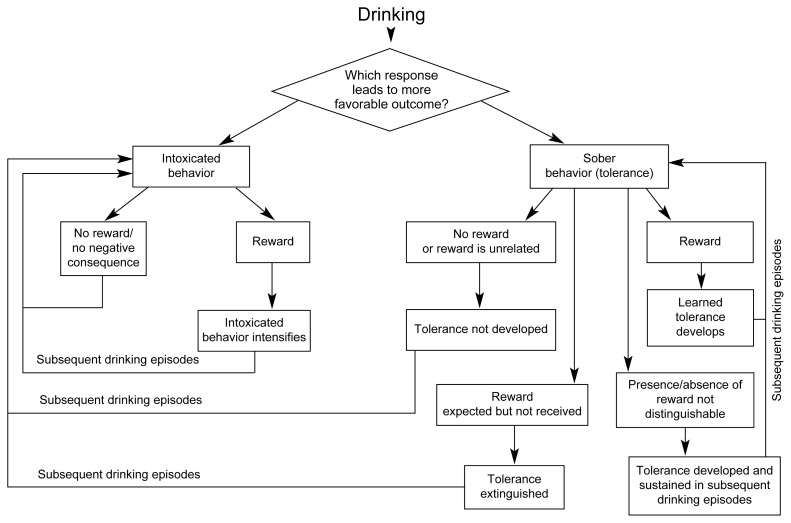
Current research suggests that drinkers will display intoxicated or sober (i.e., alcohol-tolerant) behavior following alcohol consumption, depending on which type of behavior they believe will lead to a more favorable outcome (i.e., a reward or positive consequence). The actual outcome will then influence the drinker’s behavior following subsequent drinking episodes.

Taken together, evidence on the acquisition, extinction, and retention of alcohol tolerance in social drinkers indicates that environmental outcomes of alcohol-induced behavior are important predictors of the degree of behavioral tolerance that social drinkers will display (see flowchart in figure 2). These results can be explained by associative learning: When a reliable association exists between a behavioral response and a particular outcome, people learn to expect the response to yield the same outcome each time. Applying this interpretation to the evidence described thus far suggests that drinkers display greater tolerance in situations in which they expect sober behavior to yield the most favorable outcome.

This conclusion also suggests that rewarding other types of behavior after drinking likewise would increase the occurrence of those behaviors. For example, a drinking situation in which flagrant intoxication is rewarded would be expected to lead to an intensification of behavioral impairment—a prediction that recently has been confirmed (Zack and Vogel-Sprott in press). In this study, a group of subjects displayed intense impairment following repeated doses of alcohol when impaired behavior was rewarded, whereas another group displayed tolerance when the same reward was given for sober behavior. Loosely speaking, whether a drinker displays tolerance or gross impairment as a result of drinking alcohol apparently depends on which behavior he or she expects will yield the more favorable outcome. This interpretation closely parallels some learning theories that attribute the ability to adapt a goal-directed response to expectancies acquired from learned associations between the response and its outcome (e.g., Bolles 1979).

## Compensating for Alcohol Effects

Substantial research indicates that a compensatory response that counteracts the effects of a drug underlies tolerance. Various studies (e.g., Siegel 1989) have shown that animals that have developed tolerance may display a compensatory reaction when they receive a placebo in the presence of cues signaling drug administration (i.e., when they expect a drug but receive a placebo instead). In drinkers who display alcohol-tolerant behavior, some compensatory response also may be operating to counteract alcohol’s disrupting behavioral effect. A compensatory response cannot be observed directly when alcohol effects are also present, but such a response theoretically should affect a drinker’s behavior by shifting it in a direction opposite to that attributed to the effect of alcohol. For example, if a person performs a task more slowly after drinking alcohol, a compensatory response should speed performance.

**Figure 3:**
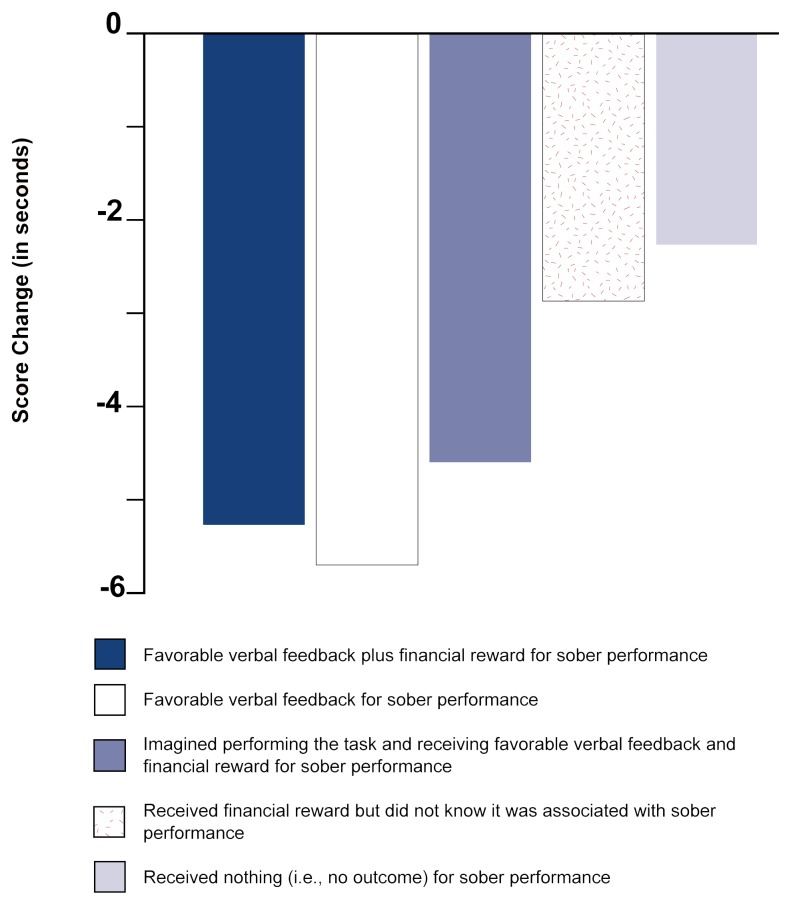
Compensation for alcohol effects. In four sessions, subjects performed or mentally rehearsed an eye-hand coordination task after consuming alcohol; their completion times (i.e., scores) were recorded. In a fifth session, the subjects performed the task but received a placebo instead of alcohol. The subjects’ compensation for the anticipated effects of alcohol was measured by comparing their average speed at finishing the task with a score recorded earlier, when they were sober. All subjects performed faster after drinking the placebo, but those who were least impaired after drinking (i.e., had become most tolerant) displayed the strongest compensation, indicated by the greatest decrease in their scores. Results imply that tolerance to alcohol involves learning to compensate for its impairing effects. NOTE: Zero = Sober baseline score. SOURCE: Adapted from Sdao-Jarvie, K., and Vogel-Sprott, M. Learning alcohol tolerance by mental or physical practice. *Journal of Studies on Alcohol* 53(6):533–540, 1992. p. 538.

Some researchers testing for a compensatory response have surreptitiously substituted a placebo for alcohol after a series of drinking sessions in which subjects who were rewarded for sober performance displayed tolerance and unrewarded control subjects did not (e.g., Sdao-Jarvie and Vogel-Sprott 1991). After drinking a placebo, all the subjects exhibited a compensatory response to some degree, as indicated by faster, improved task performance. Because they all had received their doses of alcohol and the placebo in the same environment, the expectation of receiving alcohol could have contributed to the compensatory response the study subjects displayed. Nevertheless, the groups that had received the reward and had shown the highest tolerance to alcohol displayed a much greater compensatory improvement in performance compared with the control groups (see figure 3 for results from one study). These findings are consistent with the theory that tolerance stems from a compensatory response. The findings also indicate that both the expectation of receiving alcohol and the expectation of a reward for sober performance will strengthen tolerance. (For a more technical learning-theory explanation of the contribution of these expectancies to tolerance development, see Bennett 1992.)

## Learning To Compensate for Alcohol Effects

Why should rewarding sober behavior strengthen a compensatory response? Evidence exists that a reward provides drinkers with an opportunity to learn a new behavioral strategy to compensate for the impairing effect of alcohol.

Research on motor-skill learning indicates that rewarding efficient performance provides feedback that helps the learner identify the change in behavior required to perform more skillfully (e.g., Schmidt 1988). The learner maintains the behavioral changes that yield a reward and discards those that do not. In a similar way, a drinker’s progressive development of behavioral tolerance as alcohol doses are repeated may reflect the gradual acquisition of a behavioral strategy intended to compensate for impaired behavior and maximize reward.

If a person displaying tolerance (i.e., no impairment) in one psychomotor task has learned a new behavior to overcome alcohol’s effects, the learned behavior should be transferable to a similar task performed for the first time after alcohol consumption. Research confirms such skill transfer in drinking situations that reward sober performance. When drinkers display tolerance to repeated doses of alcohol on one task, their tolerance transfers to a second task (Rawana and Vogel-Sprott 1985).

Researchers also have directly tested the idea that new learned behavior contributes to alcohol-tolerant performance. Drug-free performance of a psychomotor task can be impaired by environmental factors, such as lowered lighting or reduced visibility of a moving target. Studies have shown that behavioral tolerance to alcohol increases when subjects receive prior training to overcome impairment induced by such environmental factors (Zinatelli and Vogel-Sprott 1993; Easdon and Vogel-Sprott 1996). In these studies, all subjects first practiced a task while drug-free, although some subjects did so under environmentally induced impairment. The results showed that subjects receiving practice in overcoming environmental impairment later displayed greatly enhanced behavioral tolerance to alcohol. Apparently, practicing the task under environmentally induced impairment provided these subjects with an opportunity to learn a compensatory behavioral strategy that they could then transfer to improve their resistance to alcohol-induced impairment. These effects are analogous to cross-tolerance between two drugs; in this case, however, the cross-tolerance occurs between environmental and alcohol impairment.

Other research suggests that a technique commonly referred to as “mental rehearsal” can build tolerance as an alternative to actually performing a task after drinking alcohol (Annear and Vogel-Sprott 1985; Sdao-Jarvie and Vogel-Sprott 1986; Vogel-Sprott et al. 1984; Zinatelli and Vogel-Sprott 1990). This technique, often applied to improve motor skills in sports, involves imagining task performance before putting it into practice. To investigate the effect on tolerance of mental rehearsal under the influence of alcohol, subjects in the cited studies drank repeated doses of alcohol and either mentally rehearsed a task with an imaginary reward for sober performance or actually practiced the task and received a reward for sober performance. After the treatments concluded and all groups performed the task after drinking alcohol, both the mental-rehearsal and the task-practice groups displayed complete tolerance (i.e., no impairment) under the influence of alcohol. Thus, behavioral tolerance to alcohol apparently can be acquired either by mentally rehearsing or actually practicing sober performance after drinking alcohol.

## Conclusions and Implications

Comments and observations spanning more than a century have suggested that drinkers retain some volitional control over the behavioral effects of alcohol. Contemporary research now indicates that learned associations between alcohol-induced behavior and its environmental consequences may account for this volitional control. The important factor promoting behavioral tolerance in a drinking situation appears to be the association between the display of sober behavior and a favorable environmental outcome. Studies reviewed in this article show that drinkers acquire and display behavioral tolerance in drinking situations in which they expect sober performance to yield a positive outcome or reward. Subsequently withholding the reward so that the drinkers’ expectancy is not confirmed will extinguish their tolerance, even though they continue to drink alcohol. In contrast, drinkers retain tolerance well in drinking situations that provide no clues to indicate that a rewarding outcome is removed. Thus, the endurance of behavioral tolerance appears to depend greatly on maintaining the expectation of a favorable outcome for sober performance after drinking alcohol.

Taken together, the findings indicate that behavioral tolerance is learned. This conclusion has important implications in a variety of areas, including the following:

- Understanding behavioral tolerance. The development of behavioral tolerance to alcohol can be attributed to normal learning processes. Like any learned response, behavioral tolerance is displayed when it is reliably associated with a favorable consequence. Three exposures to alcohol under such conditions are sufficient to develop behavioral tolerance in social drinkers (Sdao-Jarvie and Vogel-Sprott 1991; Sdao-Jarvie and Vogel-Sprott 1992). Therefore, the environmental outcome expected by the drinker appears to be a far more critical determinant of behavioral tolerance than a large number of alcohol exposures is. The process of learning about the relationship between behavior and outcomes in a given situation continually occurs, and the associations that will be learned can be changed by altering the environmental consequence of a response. In other words, behavioral tolerance can be controlled by controlling its environmental consequence.
- Using behavioral tolerance to assess alcohol abuse. Many people assume that drinking must cease for tolerance to subside. Although this requirement may apply to alcohol tolerance observed at cellular and neuronal levels (i.e., physiological tolerance), a period of abstinence is not needed to reduce behavioral tolerance. Despite continued alcohol use, tolerant performance displayed by drinkers will extinguish when they do not expect or do not receive a favorable outcome. This apparent adaptive flexibility of a drinker’s behavioral response to alcohol implies that clinical assessments of behavioral tolerance are unlikely to be a reliable diagnostic symptom of a drinker’s alcohol abuse or risk of physical dependence.
- Attributing responsibility for behavior after drinking. The finding that social drinkers will display either tolerance or significant impairment, depending on which they expect to yield a more favorable consequence, suggests that these expectations also may influence other types of activities performed under the influence of alcohol, including antisocial activities. This idea has implications particularly for alcohol abusers who engage in harmful, violent, or obnoxious behavior after drinking. Like social drinkers, most alcohol abusers are not physically dependent on alcohol, and the line between the two groups can be difficult to draw. Alcohol abusers who engage in problem behavior, however, contribute to a long-standing societal problem commonly attributed to alcohol’s intoxicating chemical action. For example, in 1991 a man was acquitted on a sexual assault charge by a Quebec court judge on the grounds that the defendant probably was too intoxicated to know what he was doing when he broke into a locked home and attacked a 65-year-old, wheelchair-bound woman. Pardoning antisocial activities that occur under the influence of alcohol on the grounds that alcohol is responsible may be counterproductive, however, because it fosters the expectation of minimal or nonexistent adverse consequences for antisocial behavior. The removal of a penalizing consequence makes the expected outcome of such behavior more favorable than it would be otherwise. As a result, the undesirable behavior is potentially more likely to occur.

In summary, the research reviewed in this article suggests that the consequences drinkers learn to expect will influence their behavior. Theoretically, then, alcohol-related antisocial behavior might be reduced by cultivating the expectation that socially acceptable drinking behavior yields a more favorable outcome. This may be achieved by programs and policies that advocate responsible drinking behavior and consistently penalize antisocial behavior under the influence of alcohol.

## Footnotes

1 Throughout this article, the term “social drinker” refers to a person who consumes alcohol but does not experience any alcohol-related problems and is not alcohol dependent. The terms “alcoholic” and “alcohol abuse” as used in this article encompass all levels of problem alcohol use and do not refer to a particular diagnostic system.

2 This article presents evidence that learning plays a crucial role in the acquisition and display of behavioral tolerance to alcohol in drinkers who have no alcohol-related problems. For drinkers who have become physically dependent on alcohol, however, the story may differ. Research cited in this article still leaves open the possibility that the behavioral tolerance of alcohol-dependent drinkers involves some alcohol-induced biological changes. In other words, the processes initiating behavioral tolerance to alcohol may be entirely different from those sustaining tolerance after physical dependence has been established.
